# miRNome profiling of LSC-enriched CD34^+^CD38^−^CD26^+^ fraction in Ph^+^ CML-CP samples from Argentinean patients: a potential new pharmacogenomic tool

**DOI:** 10.3389/fphar.2020.612573

**Published:** 2021-01-11

**Authors:** María Sol Ruiz, María Belén Sánchez, Simone Bonecker, Carolina Furtado, Daniel Koile, Patricio Yankilevich, Santiago Cranco, María del Rosario Custidiano, Josefina Freitas, Beatriz Moiraghi, Mariel Ana Pérez, Carolina Pavlovsky, Ana Inés Varela, Verónica Ventriglia, Julio César Sánchez Ávalos, Irene Larripa, Ilana Zalcberg, José Mordoh, Peter Valent, Michele Bianchini

**Affiliations:** ^1^ Centro de Investigaciones Oncológicas—Fundación Cáncer, Ciudad Autónoma de Buenos Aires, Argentina; ^2^ Centro de Transplante de Medula Óssea, Instituto Nacional de Câncer, Rio de Janeiro, Brazil; ^3^ Programa de Genética, Instituto Nacional de Câncer, Rio de Janeiro, Brazil; ^4^ Instituto de Investigación en Biomedicina de Buenos Aires (IBioBA)-CONICET-Partner Institute of the Max Planck Society, Ciudad Autónoma de Buenos Aires, Argentina; ^5^ Instituto Alexander Fleming, Ciudad Autónoma de Buenos Aires, Argentina; ^6^ Hospital Nacional Posadas, Buenos Aires, Argentina; ^7^ Hospital J. M. Ramos Mejía, Ciudad Autónoma de Buenos Aires, Argentina; ^8^ Hospital Interzonal General de Agudos, Buenos Aires, Argentina; ^9^ Fundaleu, Ciudad Autónoma de Buenos Aires, Argentina; ^10^ Instituto de Medicina Experimental, CONICET/Academia Nacional de Medicina, Ciudad Autónoma de Buenos Aires, Argentina; ^11^ Centro de Investigaciones Oncológicas, Fundación Cáncer, Buenos, Aires, Argentina; ^12^ IIBBA-CONICET, Fundación Instituto Leloir, Buenos, Aires, Argentina; ^13^ Instituto Alexander Fleming, Buenos, Aires, Argentina; ^14^ Division of Hematology and Hemostaseology, Department of Internal Medicine I, Medical University of Vienna, Vienna, Austria; ^15^ Ludwig Boltzmann Institute for Hematology and Oncology, Medical University of Vienna, Vienna, Austria

**Keywords:** microRNAs, metabolism, leukemic stem cell, leukemia, CD26

## Abstract

Chronic myeloid leukemia (CML) is a myeloid stem cell neoplasm characterized by an expansion of myeloid progenitor cells and the presence of BCR-ABL1 oncoprotein. Since the introduction of specific BCR-ABL1 tyrosine kinase inhibitors (TKI), overall survival has improved significantly. However, under long-term therapy patients may have residual disease that originates from TKI-resistant leukemic stem cells (LSC). In this work, we analyzed the miRNome of LSC-enriched CD34^+^CD38^−^CD26^+^ and normal hematopoietic stem cells (HSC) fractions obtained from the same chronic phase (CP) CML patients, and stem and progenitor cells obtained from healthy donors (HD) by next-generation sequencing. We detected a global decrease of microRNA levels in LSC-enriched CD34^+^CD38^−^CD26^+^ and HSC fractions from CML-CP patients, and decreased levels of microRNAs and snoRNAs from a genomic cluster in chromosome 14, suggesting a mechanism of silencing of multiple non-coding RNAs. Surprisingly, HSC from CML-CP patients, despite the absence of *BCR-ABL1* expression, showed an altered miRNome. We confirmed by RT-qPCR that the levels of miR-196a-5p were increased more than nine-fold in CD26^+^ (*BCR-ABL1* ^*+*^) vs. CD26^−^ (*BCR-ABL1* ^−^) CD34^+^CD38^−^ fractions from CML-CP patients at diagnosis, and *in silico* analysis revealed a significant association to lipid metabolism and hematopoiesis functions. In the light of recent descriptions of increased oxidative metabolism in CML LSC-enriched fractions, these results serve as a guide for future functional studies that evaluate the role of microRNAs in this process. Metabolic vulnerabilities in LSCs open the road for new therapeutic strategies. This is the first report of the miRNome of CML-CP CD34^+^CD38^−^ fractions that distinguishes between CD26^+^ (*BCR-ABL1* ^*+*^) and their CD26^−^ (*BCR-ABL1* ^*-*^) counterparts, providing valuable data for future studies.

## Introduction

Chronic myeloid leukemia (CML) originates from a hematopoietic stem cell (HSC) that acquires the reciprocal translocation t(9;22)(q34;q11) and thus the Philadelphia chromosome (Ph) (Rowley, 1973). The resulting fusion gene, *BCR-ABL1*, encodes an oncogenic protein with constitutive tyrosine kinase activity. Treatment of CML patients was revolutionized by the introduction of specific tyrosine kinase inhibitors (TKI), like imatinib, nilotinib or dasatinib. These TKIs effectively induce apoptosis in leukemic cells in patients with CML in chronic phase (CP) (Druker et al., 1996). However, the response of patients to TKI treatment is heterogeneous, and about 40% of imatinib-treated patients require a switch of TKI due to intolerance or resistance to treatment (Holyoake and Vetrie, 2017). Other patients with optimal response to TKI show persistence of the leukemic clone, even after several years of treatment (Chomel et al., 2011). A subset of TKI-treated CML patients can achieve a deep molecular response during therapy (Holyoake and Vetrie, 2017). However, only half of them or even less can sustain a treatment-free remission (Mahon et al., 2010; Etienne et al., 2017; Ross et al., 2018; Saussele et al., 2018).

Leukemic stem cells (LSC) are defined as a population of cells that gives rise and maintains the leukemic clone (Bonnet and Dick, 1997; Valent et al., 2012). The classical view of CML considers that LSC derive from the acquisition of BCR-ABL1 in a HSC (Nguyen et al., 2012). However, BCR-ABL1 alone is unable to induce a leukemia (Foley et al., 2013). Rather, additional molecular lesions and hits are required for full transformation of clonal pre-leukemic (stem) cells into fully malignant leukemic (stem) cells (Valent et al., 2012; Valent et al., 2013). Correspondingly, single-cell gene expression analysis revealed great heterogeneity within LSC populations (Giustacchini et al., 2017; Warfvinge et al., 2017). Moreover, the most primitive LSC population has been described as quiescent CD34^+^CFSE^max^, CD34^+^CD38^−^CD90^+^CD93^+^ or Lin^−^CD34^+^CD38^−/low^CD45RA^−^cKIT^−^CD26^+^ cells (Holyoake et al., 1999; Neviani et al., 2013; Warfvinge et al., 2017; Kinstrie et al., 2020). Their normal counterparts, HSC, also constitute a heterogeneous population, and individual HSC exhibit differences in properties related to their stem cell nature: self-renewal, quiescence, repopulation capacity, and differentiation potential (Mckenzie et al., 2006; Valent et al., 2013). The mechanisms underlying the regulation of such properties are not completely understood; however, they depend on both intrinsic (such as the levels of specific transcription factors) and extrinsic (such as signals coming from the bone marrow niche) factors (Mckenzie et al., 2006; Nakamura-Ishizu et al., 2014). In CML, most LSC and their subclones may be sensitive to TKI therapy. However, certain stem cell classes, especially pre-leukemic neoplastic stem cells may be resistant because they are slowly cycling cells and exhibit multiple forms of stem cell resistance (Valent et al., 2012; Valent et al., 2013). TKI-resistance of CML LSC has been associated to both cell-autonomous (Corbin et al., 2011; Kumari et al., 2012) and extrinsic factors (Arrigoni et al., 2018; Zhang et al., 2018; Silvestri et al., 2020). Sometimes even LSC may survive TKI therapy and thus persist in CML patients. The persistence of LSC in patients under TKI therapy has fueled intensive research on this topic, in order to identify novel therapeutic targets that enable the complete eradication of the leukemic clone in all patients (Vetrie et al., 2020). On the other hand, it is not clear whether the heterogeneity observed in the LSC population is related to different responses to TKI treatment.

In CML, recent reports have characterized the transcriptome (Bruns et al., 2009; Giustacchini et al., 2017; Kinstrie et al., 2020), protein networks (Abraham et al., 2016), and the metabolome of stem/progenitor fractions in CML (Kuntz et al., 2017). Gene expression profiling of the CD34^+^CD38^−^ fraction (which includes quiescent but mostly non-quiescent LSC) in CML patients revealed a transcriptional profile resembling normal CD34^+^ myeloid progenitor cells, with decreased levels of transcription factors involved in maintenance of stem-cell fate, suggesting loss of quiescence (Bruns et al., 2009). Single-cell RNA sequencing revealed an enrichment of gene sets related to mechanistic target of rapamycin kinase (MTOR), targets of E2F transcription factors, G2/M checkpoints, oxidative phosphorylation, and glycolysis-associated gene expression in *BCR-ABL1* ^*+*^ stem cells (Giustacchini et al., 2017). However, little is known about microRNA-mediated regulation of gene expression in this population. MicroRNAs are small (19–25 nt), non-coding RNAs that can regulate multiple targets, mainly by mRNA destabilization or inhibition of protein translation. They are evolutionary conserved and have shown to be relevant for multiple physiological and pathological processes (Calin and Croce, 2006). One report has shown the involvement of microRNAs in TKI sensitivity in CML LSC (Salati et al., 2017). Recent evidence has shown that miR-300 is a tumor suppressor microRNA able to induce quiescence in CML LSCs (Silvestri et al., 2020), and that miR-126-3p regulates quiescence, self-renewal and engraftment capacity of CML LSCs (Zhang et al., 2018). Advances in the characterization of aberrant expression of surface markers have allowed the prospective isolation of LSC and HSC from CML patients (Herrmann et al., 2014; Warfvinge et al., 2017). In this work, we characterized the miRNome of LSC-enriched CD34^+^CD38^−^CD26^+^ fraction (*BCR-ABL1* ^+^) and its *BCR-ABL1* ^−^ counterpart (CD34^+^CD38^−^CD26^−^ fraction, defined as “CML-CP HSC” in this article) isolated by fluorescence-activated cell sorting (FACS) from CML-CP patients at diagnosis by small RNA-Next-Generation Sequencing (NGS), in order to identify differential molecular mechanisms that contribute to unravel LSC biology, and the possible therapeutic implications of such differences. We observed a global decrease in microRNA levels in LSC-enriched and HSC fractions from CML-CP patients in comparison with HSC obtained from healthy donors (HD). Surprisingly, compared to HSC from HD, we detected decreased levels in the LSC-enriched fraction of microRNAs and snoRNAs belonging to a genomic cluster located in chromosome 14 (14q32) that contains imprinted genes, suggesting an epigenetic mechanism of silencing of multiple non-coding RNAs. Bioinformatic analysis of microRNAs with altered levels in LSC revealed a significant association with lipid metabolism and hematopoiesis. Finally, we confirmed an increase in the levels of miR-196a-5p in LSC-enriched CD34^+^CD38^−^CD26^+^ fraction by reverse transcription followed by quantitative PCR (RT-qPCR) in additional CML-CP patients. Our results suggest that some microRNAs may act as mediators of the dysregulated metabolism observed in CML stem/progenitor fractions, and opens an exciting pathway for future research.

## Materials and Methods

### Patient Samples

The project was approved by the Institutional Review Board, at Instituto Alexander Fleming (Buenos Aires, Argentina). All procedures involving human participants were in accordance with the ethical standards of the institutional research committee and with the 1964 Helsinki declaration and its later amendments. All patients and HD gave written informed consent. Bone marrow (BM) or peripheral blood (PB) samples were obtained from newly diagnosed, untreated CML-CP patients. Patient samples used for library preparation for small RNA-NGS and validation by RT-qPCR are listed in Table 1. Mononuclear cells (MNC) were isolated by density-gradient centrifugation (Ficoll-Paque PLUS, GE Healthcare Life Sciences) for 30 min at 400 × *g*, followed by one wash in phosphate buffered saline (PBS, GIBCO), a red cells lysis step, and a low-speed centrifugation step (12–15 min at 200 × *g*) for removal of the platelet-rich fraction. Up to 2 × 10^8^ MNC were used for isolation of CD34^+^ cells.

**TABLE 1:** CML-CP and HD samples used for small RNA-NGS and validation by RT-qPCR.

Code	Type	Sex	Age	No. events in HSC fraction	No. events in LSC-enriched fraction	No. events in progenitor fraction
CML-CP samples used for small RNA-NGS (pooled)						
N26	BM	M	24	6,896	1,857	ND
N33	PB	M	54	1,859	0	ND
IM	BM	M	22	NE*	3,046	ND
HD samples used for small RNA-NGS (pooled)						
2891	Buffy coat	M	36	1,578	N/A	7,624
2890	Buffy coat	F	31	1,316	N/A	13,724
2810	PB	F	50	156	N/A	ND
3060	PB	F	54	181	N/A	ND
3308	Buffy coat	F	37	1,700	N/A	13,000
CML-CP samples used for validation by RT-qPCR						
N36	PB	M	56	2,857	2,200	37,272
N47	PB	M	58	1,128	4,504	96,333
N55	PB	M	25	NE*	9,276	19,861
N38	PB	M	31	592	0	2,300
N46	PB	F	38	NE*	3,360	61,428
N56	PB	M	55	4,267	2,910	291,653
HD samples used for validation by RT-qPCR						
3984	Buffy coat	F	36	1,117	N/A	1,779
3308	Buffy coat	F	37	1,682	N/A	2,027
2771	Buffy coat	M	53	4,460	N/A	9,539
4532	Buffy coat	F	57	2,314	N/A	13,256

NE*, fractions not evaluated (pattern 3); ND, not done; N/A, not applicable.

The number of events refers to the number of sorted cells obtained from each fraction.

### Isolation of CD34^+^ Cells

In order to enrich for stem and progenitor cells, we performed a positive selection using CD34 MicroBeads (Miltenyi Biotech), according to manufacturer’s instructions. The CD34^+^ fraction was immediately used or cryopreserved in 1 ml of freezing medium (Supplementary Material).

### CFU Assay for Assessment of Purity in Sorted Fractions

Between 250 and 500 CD34^+^ cells were directly sorted into 250 µl of enriched methylcellulose (Methocult H4435 Medium, Stem Cell Technologies, Vancouver, Canada), and then plated into p35 culture dishes containing a final volume of 1.1 ml of enriched methylcellulose. Cells were incubated at 37°C in a humid chamber. After 14–18 days, pools of four to six colonies (CFU-GM, BFU-E and mixed CFU-GEMM) were plucked from methylcellulose, resuspended in 500 µl of Roswell Park Memorial Institute—1640 medium (RPMI-1640, GIBCO), and centrifuged. Cells were resuspended in 100 µl of lysis solution (RNAqueous-Micro Kit, Ambion), and kept at −20°C until RNA extraction was performed. Total RNA was extracted following manufacturer’s instructions, and *BCR-ABL1* mRNA was measured by RT-qPCR (Supplementary Material).

### Isolation of LSC and HSC by FACS

Total number of cells used for FACS varied according to the yield of each sample. CD34^+^ cells or MNC from CML-CP patients or HD were incubated with the following antibodies: 5 µl CD45-PerCP (2D1, BD Biosciences), 2.5 µl CD34-FITC (AC136, Miltenyi Biotech), 2.5 µl CD38-PE (IB6, Miltenyi Biotech), and 15 µl CD26-APC (FR10-11G9, Miltenyi Biotech), in a final volume of 100 µl of MACS buffer, for 15 min at room temperature. Cells were washed once with 1 ml of PBS (GIBCO) and resuspended in 300 µl of PBS. Sorting was performed in a FACS Aria II cytometer (BD Biosciences), located at Facultad de Ciencias Exactas y Naturales, Universidad de Buenos Aires (Buenos Aires, Argentina). Setting of positive and negative gates for CD38, CD34, and CD26 was performed on the CD45^low^ population; therefore, isotype control tube included 2.5 µl Mouse IgG2a-FITC (Miltenyi Biotech), 2.5 µl Mouse IgG2a-PE (Miltenyi Biotech), 15 µl Mouse IgG2a-FITC (Miltenyi Biotech), and 5 µl CD45-PerCP. In order to avoid electronic aborts that could affect the purity of sorted fractions, the parameter “window extension” was set to zero. Other parameters included 70 µm nozzle, and “purity.” Cells were collected in aseptic conditions, directly into 100 µl of lysis buffer for RNA extraction (RNAqueous-Micro Kit, Ambion), in RNAse-free 200 µl tubes, or in enriched methylcellulose for assessment of purity. Flow-cytometry data analysis was performed with BD FACSDiva (version 6.1.3) and FlowJo (version 7.6.2) software.

Total RNA containing small RNAs (<200 nt) was extracted following the protocol from RNAaqueous-micro kit (Ambion) with a slight modification: 125 µl of EtOH 100% were added to the lysate and vortexed; the rest of the protocol was performed according to manufacturer’s instructions. RNA elution was performed twice (9 µl each) using pre-warmed distilled water (75°C). RNA was kept at −80°C. Quality and conservation of the small RNA fraction were assessed with Agilent 2100 Bioanalyzer (total RNA Nano kit), at Fundación Instituto Leloir (Buenos Aires, Argentina).

### Concentration of Pooled Samples for Small RNA-NGS

RNAs extracted from different samples were combined in order to increase RNA yield before NGS library preparation. After mixing, RNA was freezed at −80°C, and transported from Argentina to Brazil in dry ice. Samples were concentrated using a vacuum centrifuge and resuspended in 7 µl of distilled water (5 min at 50°C). Quantification of RNA was performed using Qubit 2.0. The entire content was used for library preparation (<140 ng for LSC-enriched, CML-CP HSC and HD HSC fractions, and 210 ng for HD progenitor fraction).

### Preparation of Libraries for Small RNA-NGS in HiSeq 2500 (Illumina)

Libraries from each pool of samples (CML-CP LSC-enriched CD34^+^CD38^−^CD26^+^, CML-CP HSC CD34^+^CD38^−^CD26^−^, HD HSC CD34^+^CD38^−/dim^, HD progenitors CD34^+^CD38^+^) were prepared using Truseq Small RNA kit (Illumina), following manufacturer’s instructions (15 PCR cycles). The protocol is based on the selective ligation of RNAs with free 3′OH and 5′phosphate ends, resulting from precursor cleavage during small RNA biogenesis (Bartel, 2018). Therefore, other small RNAs besides microRNAs are included in the library: fragments of tRNAs, small nucleolar RNAs (snoRNAs), small nuclear RNAs (snRNAs), and piwiRNAs. Estimated size of the libraries was 147–157 bp, which were purified by band excision after polyacrylamide denaturing gel electrophoresis (Novex 6% TBE, Invitrogen). Quantification of libraries was performed by qPCR (KAPA SYBR, Roche Life Sciences). Libraries were concentrated before sequencing by vacuum centrifugation. Single-end sequencing was performed on HiSeq 2500 (Illumina) at Instituto Nacional de Câncer (Rio de Janeiro, Brazil).

### Bioinformatic Analysis of Small RNA-NGS

Low quality lectures were filtered (fastq_quality_filter; >80% reads with Q > 20), and contaminant sequences were removed (3′ and 5′ adaptors, indexes). Identification of known microRNAs was performed with Chimira (Vitsios and Enright, 2015); raw microRNA counts from each pool of samples is available (Supplementary Data Sheet S2). Differential expression analysis was performed using GFOLD algorithm (*c* = 0.01), which is especially suited for experiments without biological replicates, after mapping against a database of snoRNA/miRNA (HISAT2) (Feng et al., 2012). Complete results from GFOLD analysis are available (Supplementary Data Sheet S1). Traditional analysis of potential targets and related pathways was performed using miRPath (*Diana tools*) (Vlachos et al., 2015), and ChemiRs (Su et al., 2016). The parameters used for miRPath analysis were: “*KEGG analysis*,” *Tarbase* (database of experimentally validated interactions), or *microT-CDS* in those cases with no experimental evidence, “*Pathway union*,” “*p-value threshold:* 0.001,” “*MicroT threshold*: 0.8,” “*Enrichment analysis method = Fisher’s exact test (hypergeometric distribution)*,” “*FDR correction* (*Benjamini & Hochberg*)”, “*Conservative stats*.” Network analysis were performed with miRNet 2.0 (Chang et al., 2020) (query by lists of microRNAs; targets databases: Genes miRTarBase v8.0/lncRNAs, degree cutoff: 1.1). Overrepresentation analysis were performed with TAM 2.0 (Li et al., 2018). Venn diagrams were created with BioVenn (Hulsen et al., 2008). Intersections between lists of microRNAs or targets were performed with R software (v.3.4.0).

### Detection of microRNAs by RT-qPCR

We evaluated the following fractions from CML-CP patients or HD samples: CML-CP LSC-enriched CD34^+^CD38^−^CD26^+^, CML-CP HSC CD34^+^CD38^−^CD26^−^, CML progenitors CD34^+^CD38^+^, HD HSC CD34^+^CD38^−/dim^, HD progenitors CD34^+^CD38^+^. We applied a modification of the protocol reported by Chen et al., based on a reverse transcription (RT) using gene-specific stem-loop primers (Chen et al., 2005) (incubation times were modified as detailed below), followed by individual qPCR reactions for each microRNA using an intercalating agent. qPCR used a specific forward primer and a universal reverse primer designed to hybridize with the constant region included in the stem-loop primer. Two multiplex RT reactions for microRNAs (M1 and M2) (Supplementary Table S1) were performed for each sample. Additionally, each sample was reverse transcribed with random primers in a separate reaction, in order to measure snRNA U6 as a reference gene for qPCR. Final concentrations of components of RT reaction were: dNTPs 0.25 mM (Invitrogen or INBIO Highway); DTT 10 mM (Invitrogen); Superscript II 2.5 U/µl (Invitrogen); RNAse inhibitor 0.2 U/µl (RNAseOUT, Invitrogen); *stem-loop* primer 0.05 µM (each) or random primers 0.01 μg/μl (Invitrogen). Incubation times were: 5 min of RNA, H_2_O, and dNTPs at 65°C; tubes were immediately placed on ice; the remaining components were added to the tube and incubated for 30 min at 16°C, 40 cycles (30 s at 30°C + 30 s at 42°C + 1 s at 50°C), followed by a final step of 5 min at 85°C. cDNA was diluted (1/2) with distilled water and stored at −20°C. Two microliters of diluted cDNA was used for each qPCR reaction, using the following conditions: forward primer 0.3 µM; universal reverse primer 0.3 µM, and SYBR Green (PowerUp SYBR Green MasterMix, Applied Biosystems; according to the information provided by the manufacturer, Mg^2+^ concentration can vary between 4.76 and 6.44 mM); incubated for 2 min at 50°C, 2 min at 95°C, 50 cycles (15 s 95°C + 1 min at 60°C), in a Rotor-Gene Q qPCR equipment (Qiagen). Melting curves were evaluated in order to assess specificity of the reaction. Quantifications were performed in duplicate. In cases were duplicate measurements differed (∆Ct > 2), a triplicate measurement was performed. RT-qPCR efficiency was estimated by performing curves of RNA; formula used for efficiency estimation was *E* = [10^(−1/*m*)^]^−1^, *m* being the slope of the curve. Sequences of all primers used for quantification of microRNAs are available (Supplementary Material).

### Statistical Analysis and Graphical Tools

GraphPad Prism 6, Microsoft Excel 2007, and Inkscape 0.92 software were used for graphics. Infostat v.2018e software (Córdoba, Argentina) was used for statistical analysis. Data from quantification of microRNAs by RT-qPCR were analyzed using the variable dCt = (Ct microRNA X − Ct snRNA U6), with a linear mixed-effects model (ANAVA): fraction (LSC-enriched, CML-CP HSC, CML-CP progenitors, HD HSC, HD progenitors) was set as a fixed effect, and sample (each patient or HD) was set as a random effect (correlation factor: compound symmetry). Variance was modeled using “VarIdent” function (using the variable “fraction”). False discovery rate was considered by multiplying *p*-values by the number of total microRNAs evaluated. *A posteriori* comparisons were performed using DCG formula (di Rienzo et al., 2011).

## Results

### Global Patterns in the miRNome of LSC-Enriched and CML-CP HSC Fractions

**FIGURE 1:**
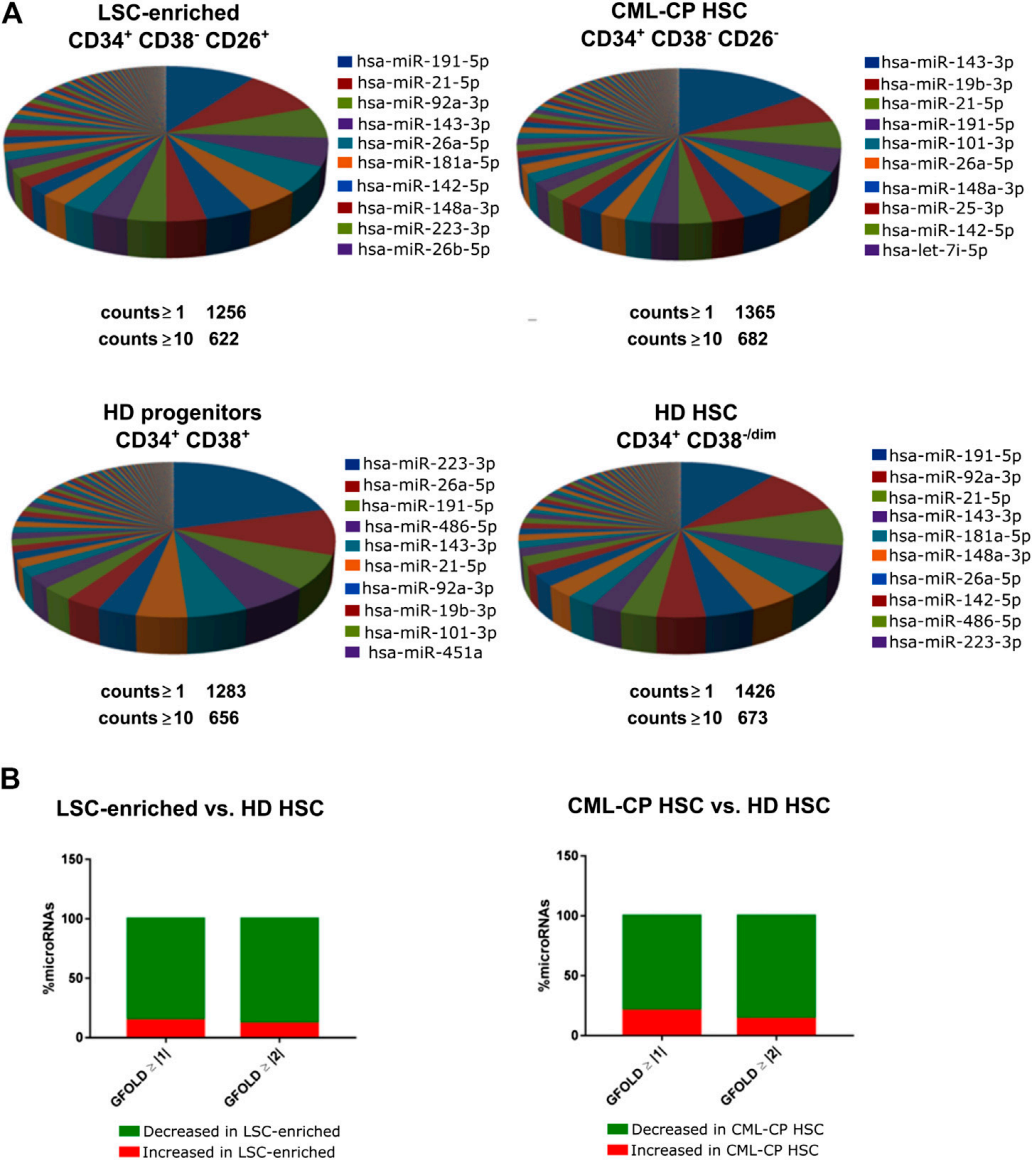
Global patterns in the miRNome of LSC-enriched CD34^+^CD38^−^CD26^+^ and CML-CP HSC fractions. **(A)** Pie chart representing the relative abundance of each microRNA in each fraction assessed by small RNA-NGS. The total number of different microRNAs (with at least 1 or 10 counts) is detailed below the pie chart. The names of the top-10 most abundant microRNAs in each fraction are detailed. **(B)** Global decrease in microRNA levels in LSC-enriched CD34^+^CD38^−^CD26^+^ or CML-CP HSC fractions compared to HD HSC. Most microRNAs dysregulated in both fractions from CML-CP samples had decreased levels (GFOLD ≥ |1| or GFOLD ≥ |2|) compared to HD HSC.

We isolated highly pure LSC-enriched and HSC fractions from CML-CP patients at diagnosis or from HD, based on a combination of cell surface markers (CD34, CD38, CD45, CD26) and flow cytometry parameters (FSC, SSC) (Supplementary Figure S1). Some patients showed no clear separation of CD26^−^ and CD26^+^ populations (Supplementary Figure S2): in those cases, both fractions included leukemic *BCR-ABL1* ^+^ cells, therefore, we only used CD26^+^ fraction. Purity was assessed by *BCR-ABL1* mRNA detection in CFU-derived colonies (Supplementary Figure S2). We extracted total RNA containing the small RNA fraction (<200 nt) from sorted cells; given that individual patient-derived fractions had low yields of RNA, samples from different patients or HD were pooled before preparation of libraries for small RNA-NGS (CML-CP LSC-enriched CD34^+^CD38^−^CD26^+^, CML-CP HSC CD34^+^CD38^−^CD26^−^, HD HSC CD34^+^CD38^−/dim^, HD progenitors CD34^+^CD38^+^). More than 1,000 (≥1 count) or 600 (≥10 counts) different microRNAs were detected in each fraction, with high abundance of a few specific microRNAs: top-10 most abundant microRNAs represented 57–69% of total microRNAs in CML-CP and HD (Figure 1A). Surprisingly, the pattern of most-abundant microRNAs showed more differences between fractions from CML-CP patients (LSC-enriched and CML-CP-HSC) than between LSC-enriched and HD-HSC fractions. Most (>80%) microRNAs dysregulated (GFOLD ≥ |1|) in LSC-enriched and HSC fractions from CML-CP patients had decreased levels compared to primitive (CD34^+^CD38^−/dim^) cells from HD, suggesting a global pattern of microRNA downregulation (Figure 1B).

### microRNAs With Altered Levels in LSC-Enriched vs. HSC Fractions From CML-CP Patients and HD

**FIGURE 2:**
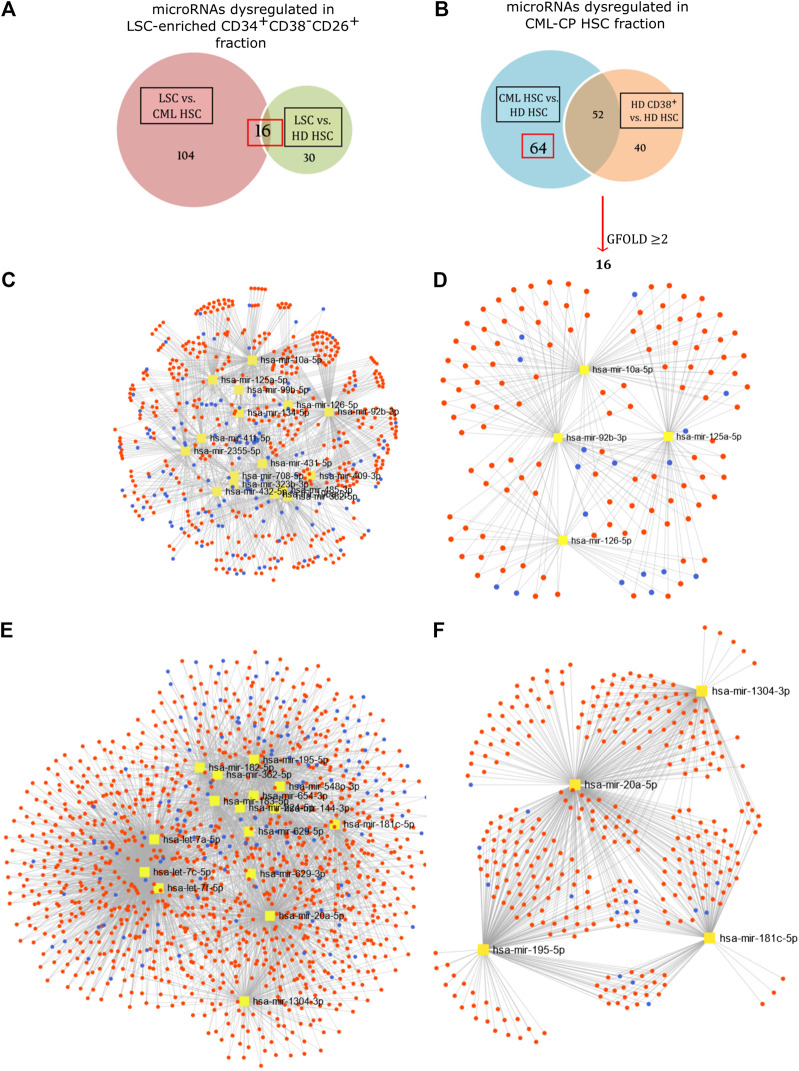
Network analysis. Differentially expressed microRNAs in LSC-enriched CD34^+^CD38^−^CD26^+^ **(A,C)** and CML-CP HSC **(B,E)** fractions were analyzed as part of a network of microRNAs (yellow), target mRNAs (red) and lncRNAs (blue) using the bioinformatic tool miRNet (v2.0). Statistically significant modules were extracted for microRNAs dysregulated in LSC-enriched CD34^+^CD38^−^CD26^+^ **(D)** and CML-CP HSC **(F)** fractions.

Differential expression of microRNAs was assessed by calculation of a GFOLD value, which is a robust fold-change parameter that considers both the absolute number and the relative difference in microRNA levels between samples. With a cut-off value of GFOLD ≥ |1|, we found 120 microRNAs dysregulated between LSC-enriched and putative HSC fractions from CML-CP patients; and 46 microRNAs between LSC-enriched and HD-HSC fractions. The intersection of both lists resulted in 16 microRNAs (Figure 2A; Supplementary Table S2). Traditional *in silico* enrichment analysis of both predicted and experimentally validated targets showed a noteworthy proportion of false positives (Supplementary Results, Supplementary Figures S3 and S4, Supplementary Table S3).

Given the observed *bias*, and the difficulty of analyzing microRNA-related pathways through their mRNA targets due to the existence of “multiple-to-multiple relationships” (a given microRNA can regulate multiple genes, and a given gene can be regulated by multiple microRNAs), we performed a network analysis with the recently updated tool miRNet (v.2.0) (Chang et al., 2020). This tool allows the simultaneous inclusion of microRNA interactions with mRNAs, transcription factors (TF), lncRNAs, small non-coding RNAs and circular RNAs. We also applied an overrepresentation analysis based on microRNA-sets using TAM 2.0, a manually curated database of functional and disease associations of microRNAs (Li et al., 2018). *Bias* assessment using the same random lists resulted in none statistically significant associations (Supplementary Data Sheet S3). Using as input microRNAs upregulated in CML-CP LSC-enriched fraction, this analysis detected a significant enrichment in “lipid metabolism” (False Discovery Rate, FDR: 0.0141), “hematopoiesis” (FDR: 0.0217), TF *Early growth response 1* (*EGR1*) (FDR: 0.007), and miR-99b cluster (FDR: 0.0138) (Table 2). Network analysis allowed the extraction of statistically significant modules (Figures 2C,D), suggesting the existence of mechanisms that regulate microRNA levels in a coordinated fashion.

**TABLE 2:** Overrepresented associations in differentially expressed microRNAs (TAM 2.0).

Category	Term	FDR	microRNA
Input: Upregulated microRNAs in LSC-enriched CD34^+^CD38^−^CD26^+^ vs. CML-CP HSC fractions (miR-92b, miR-196a, miR-126, miR-125a, miR-2355, miR-99b, miR-411, miR-10a)			
Function	Lipid metabolism	0.0141	hsa-mir-125a, hsa-mir-126, hsa-mir-196a-2, hsa-mir-196a-1
Function	Hematopoiesis	0.0217	hsa-mir-125a, hsa-mir-126, hsa-mir-196a-2, hsa-mir-196a-1
TF	EGR1	0.00704	hsa-mir-125a, hsa-mir-99b, hsa-mir-10a, hsa-mir-92b
Cluster	hsa-mir-99b cluster	0.0138	hsa-mir-99b, hsa-mir-125a
Input: downregulated microRNAs in LSC-enriched CD34^+^CD38^−^CD26^+^ vs. CML-CP HSC fractions (miR-708, miR-431, miR-134, miR-485, miR-409, miR-323b, miR-432, miR-382)			
Cluster	hsa-mir-379 cluster	0.00248	hsa-mir-382, hsa-mir-134, hsa-mir-485, hsa-mir-323b, hsa-mir-409
Input: downregulated microRNAs in CML-CP HSC vs. HD HSC (excluding let-7 family) (miR-20a, miR-195, miR-654, miR-224, miR-144, miR-181c, miR-548o, miR-182, miR-183)			
Function	Glucose metabolism	0.0514	hsa-mir-20a, hsa-mir-195, hsa-mir-144
Transcription factor	E2F1	0.0457	hsa-mir-20a, hsa-mir-224, hsa-mir-195
Transcription factor	STAT5	0.0557	hsa-mir-195, hsa-mir-20a

FDR, false discovery rate.

Only statistically significant results are shown.

**FIGURE 3:**
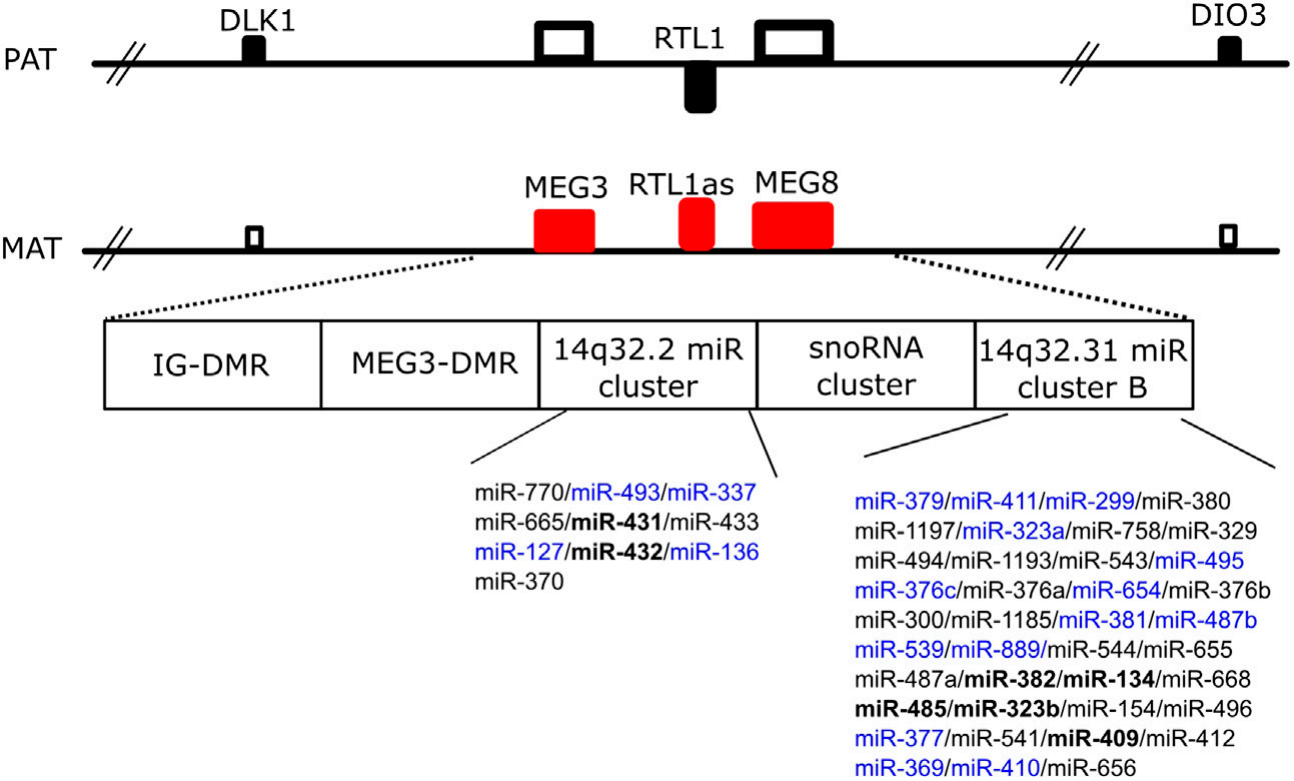
Schematic representation of the genomic 14q32 region. This region includes coding genes with paternal (PAT) imprinting (*DLK1*, *RTL1*, *DIO3*), non-coding genes with maternal (MAT) imprinting (*MEG3*, *RTL1as*, *MEG8*), two clusters of microRNAs, and one cluster of snoRNAs. microRNAs in bold showed decreased levels in LSC-enriched CD34^+^CD38^−^CD26^+^ vs. both HD HSC and CML-CP HSC; while microRNAs in blue showed decreased levels in LSC-enriched CD34^+^CD38^−^CD26^+^ vs. HD HSC but did not vary compared to CML-CP HSC. IG, intergenic; DMR, differentially methylated regions. Adapted from (Nadal et al., 2014).

Surprisingly, most (seven out of eight) microRNAs with decreased levels in LSC-enriched fraction belong to a genomic cluster located in region 14q32 (*DKL1/DIO3* locus). Moreover, inspection of microRNAs and snoRNAs from this locus revealed that 18 additional microRNAs and five snoRNAs had decreased levels in LSC-enriched vs. HD-HSC fractions (Figure 3; Supplementary Table S4). Accordingly, overrepresentation analysis by TAM 2.0 detected a significant enrichment in miR-379 cluster (located in the *DKL1/DIO3* locus) in microRNAs downregulated in the LSC-enriched fraction (FDR: 0.002, Table 2). Given that this region contains imprinted genes (Benetatos et al., 2013), this result suggests a mechanism of epigenetic silencing in the LSC-enriched CD34^+^CD38^−^CD26^+^ fraction.

### microRNAs in HSC From CML-CP Patients Show a Dysregulated miRNome Despite the Absence of BCR-ABL1

Based on the hypothesis that HSC present in CML-CP patients are not equivalent to HSC in HD, we compared microRNAs between both fractions. We found 64 microRNAs significantly dysregulated (Figure 2B); further selection (GFOLD ≥ |2|) resulted in a list of 16 microRNAs (Supplementary Table S5). It is important to clarify that HSC from HD were sorted using a less strict gating of CD38-negative cells (resulting in a CD38^−/dim^ population), because we obtained very low yields from individual samples. Therefore, in order to exclude differentially expressed microRNAs related to the inclusion of a CD38^dim^ population in HD, we excluded microRNAs that were differentially expressed between CD38^−/dim^ and CD38^+^ fractions from HD, under the assumption that some of these microRNAs would be related to the process of hematopoietic differentiation. Network (miRNet) and overrepresentation analysis by TAM 2.0 were performed. MicroRNAs belonging to Let-7 family were not included in the analysis by TAM 2.0 due to their association to multiple functions. Overrepresentation analysis resulted in “glucose metabolism” (FDR: 0.0514), TFs *E2F transcription factor 1* (*E2F1*, FDR: 0.0457) and *Signal transducer and activator of transcription 5* (*STAT5*, FDR: 0.0557) in those microRNAs downregulated in CML-CP HSC (Table 2). Network analysis allowed extraction of statistically significant modules (Figures 2E,F). These results suggest that putative HSC from CML-CP patients display an altered repertoire of microRNAs, as a consequence of either extrinsic factors (i.e., a niche altered by coexistence with leukemic cells), and/or that they are pre-leukemic neoplastic stem cells and thus harbor early, BCR-ABL1-independent genetic or epigenetic alterations that affect microRNA levels (i.e., mutations in microRNA-processing machinery).

### Validation by RT-qPCR in a New Cohort of CML-CP Patients and HD

As other techniques, NGS is not free of intrinsic bias, mainly related to library preparation, platform used for sequencing, and data analysis (Baran-gale et al., 2015). In addition to perform a technical validation, we aimed to validate NGS results in a new cohort of patients and HD using RT-qPCR. We performed a multiplex RT step that allowed us to measure individual microRNA levels using very low inputs of RNA; therefore, pooling of samples from different patients or HD was not necessary, and we could assess intra-group variability. We evaluated the following fractions from CML-CP patients or HD samples: CML-CP LSC-enriched (CD34^+^CD38^−^CD26^+^), CML-CP HSC (CD34^+^CD38^−^CD26^−^), CML-CP progenitors (CD34^+^CD38^+^), HD HSC (CD34^+^CD38^−/dim^), HD progenitors (CD34^+^CD38^+^).

**FIGURE 4:**
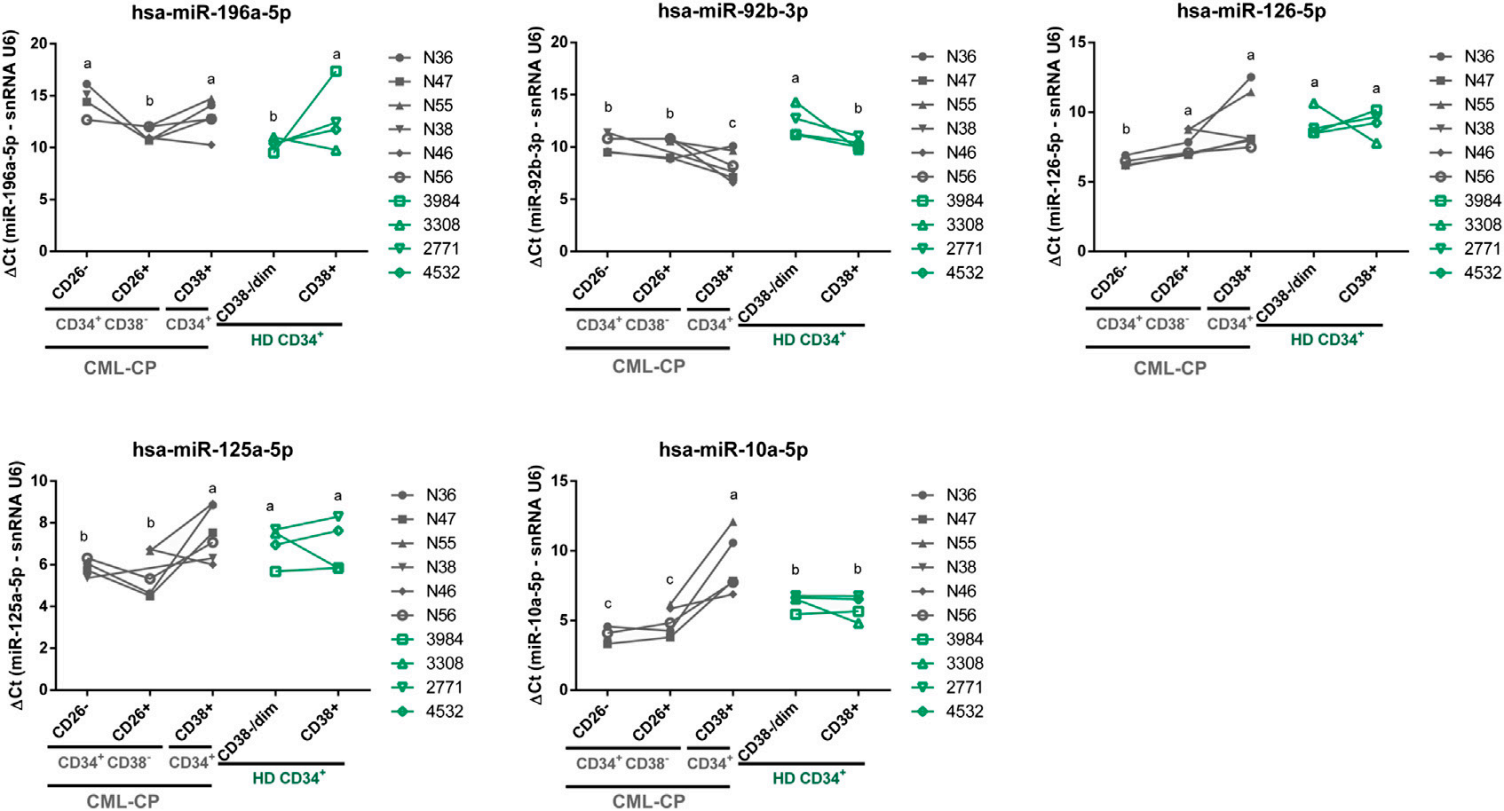
Validation of microRNAs by RT-qPCR in a new cohort of CML-CP and HD samples. Results are expressed as ΔCt = Ct (microRNA) − Ct (snRNA U6). Each dot is the mean of technical duplicates from each patient or HD. CML-CP samples are represented in grey symbols, and HD samples in green symbols. Lines connect different fractions from the same patient or HD. Different letters indicate statistically significant differences (linear mixed-effects model, *a posteriori* comparison, global α = 0.05).

We selected six microRNAs upregulated in LSC-enriched vs. HSC fractions from CML-CP patients (miR-125a-5p, miR-10a-5p, miR-126-5p, miR-92b-3p, miR-196a-5p, miR-2355-5p), because of their high GFOLD values and clustering in pathway analysis, and measured their levels in six CML-CP patients, and four HD. We also included four additional small RNAs: one potential “novel” microRNA that emerged from NGS data (“novel-3”), and three additional microRNAs which were of interest in this population according to previous references (let-7a-5p, miR-132-3p, miR-182-5p). Purity of fractions was assessed by RT-qPCR of *BCR-ABL1* in RNA isolated from sorted cells (Supplementary Figure S5). miR-2355-5p and miR-182-5p were not detected in most fractions, therefore we excluded them from posterior analysis. We detected significant differences among fractions for miR-125a-5p, miR-10a-5p, miR-126-5p, miR-92b-3p, and miR-196a-5p (global *p*-value < 0.05; linear mixed-effects model) (Figure 4). We did not detect global differences in the levels of “novel-3,” miR-let-7a-5p, and miR-132-3p (global *p*-value > 0.05; linear mixed-effects model) (Supplementary Figure S6). Only for miR-196a-5p the trend of change between CML-CP LSC-enriched and CML-CP HSC fractions was the same in NGS (fold-change LSC-enriched/CML-CP HSC = 9.6) and RT-qPCR (mean = 10.7, SD = 4.6) (Supplementary Figure S7). In contrast, differences in Ct values between CML-CP LSC-enriched and CML-CP HSC fractions were not statistically significant for miR-125a-5p and miR-92b-3p, but both were significantly increased compared to HD HSC (adjusted *p*-value < 0.05, *a posteriori comparison*, Figure 4) On the contrary, miR-126-5p and miR-10a-5p displayed opposite trends in NGS and RT-qPCR (Supplementary Figure S7).

CML-CP progenitors were only measured by RT-qPCR; interestingly miR-92b-3p and miR-10a-5p levels were significantly increased or decreased, respectively, in CML-CP progenitors in comparison with all the other CML-CP and HD fractions (Figure 4; Supplementary Figure S8 for log2-fold change values).

According to significant functions detected by TAM 2.0, we searched for possible targets of miR-196a-5p included in gene lists classified in Reactome as “Metabolism of lipids” and Gene Ontology “hematopoietic stem cell differentiation”. The search of potential targets included experimentally validated microRNA-mRNA interactions (assessed by ChemiRs) (Supplementary Figure S9). This analysis reduced the number of potential targets from 307 to 14 genes.

## Discussion

To our knowledge, this is the first description of the miRNome of CML-CP LSC-enriched CD34^+^CD38^−^CD26^+^ fraction and its CD26^−^ counterpart. In these analyses, major differences were found when analyzing *BCR-ABL1* ^+^ and *BCR-ABL1* ^−^ fractions in newly diagnosed patients. In addition, we detected several major differences in the miRNome pattern when comparing with HSC of HD. First, we observed a global downregulation of microRNAs in CML-CP LSC-enriched CD34^+^CD38^−^CD26^+^ fraction and its CD26^−^ counterpart in comparison with HSC of HD. Second, clusters and TF-associated networks of microRNAs were detected among differentially expressed microRNAs, suggesting that mature levels of functionally related microRNAs are (dys) regulated by common mechanisms. Third, compared to HSC from HD, we detected decreased levels in the LSC-enriched fraction of microRNAs and snoRNAs belonging to a genomic cluster located in chromosome 14 (14q32). Fourth, a high number of microRNAs were differentially expressed between putative HSC from CML-CP patients and HSC from HD, suggesting an altered phenotype of the “normal” HSC fraction in CML-CP patients. Finally, we confirmed by RT-qPCR that the levels of miR-196a-5p were increased more than nine-fold in LSC-enriched CD34^+^CD38^−^CD26^+^ (*BCR-ABL1* ^*+*^) vs. CD26^-^(*BCR-ABL1* ^−^) fractions from CML-CP patients at diagnosis, and *in silico* analysis revealed a significant association to lipid metabolism and hematopoiesis functions.

It is important to consider the limitations of this study in the interpretation of the results. In particular, the possible effects of pooling different samples (e.g., low statistical power or dilution of differences), and the transport between countries for small RNA-NGS (e.g., possible degradation of some microRNAs). In addition, future studies using a higher number of samples are desirable to confirm these observations. The measurement of a new cohort, non-pooled patient and HD samples in the validation step by RT-qPCR partially overcame these limitations.

Global downregulation of microRNAs in cancer has been reported in different tumors (Lu et al., 2005). Multiple mechanisms have been described to explain microRNA dysregulation in cancer, including genomic structural variations, altered regulation of microRNA transcription, epigenetic changes, defective microRNA processing machinery, and dysregulation of the complex that mediates pre-microRNA export from the nucleus (Croce, 2009). In CML, global microRNA depletion in patient samples has not been reported so far. In the work of Zhang et al., they showed, in K562 cells and CML CD34^+^ cells, that BCR-ABL1 can affect the export of miR-126 precursors from the nucleus to the cytoplasm, through phosphorylation of SPRED1, a negative regulator of RAS superfamily of proteins, interfering with Ran-exportin 5-RCC1 complex (Zhang et al., 2018). Interestingly, this effect was reversible by treatment with Nilotinib. However, in our work, we observed decreased levels of mature microRNAs in both *BCR-ABL1* ^*+*^ and *BCR-ABL1* ^−^ primitive (CD34^+^CD38^−^) cells compared to HSC from HD, suggesting a BCR-ABL1-independent mechanism.

Clustering of microRNAs dysregulated in the LSC-enriched fraction suggests the existence of mechanisms of coordinated regulation. MicroRNAs can belong to families in which members are evolutionary related, therefore they share regions of common sequences, and can regulate similar or related targets. In this context, miR-125a and miR-10a belong to the miR-10/miR-100 family, and we found a significant positive correlation of both microRNAs in samples evaluated by RT-qPCR [r (Pearson) = 0.85; *p* = 5.7 × 10^−7^]. This suggests that future studies aimed at evaluating the functional relevance of microRNAs dysregulated in this system should take into consideration possible functional redundancy between related microRNAs. In fact, knockout experiments of microRNAs belonging to the same family have shown partially redundant effects on mice (Wong et al., 2015). Therefore, the combination of individual and simultaneous knockdown of correlated microRNAs would be an ideal approach.

In humans, the *DKL1/DIO3* locus at the 14q32 region, contains the paternally expressed genes *Delta-like 1 homolog* (*DLK1*), *Retrotransposon-like 1* (*RTL1*), and *Iodothyronine deiodinase 3* (*DIO3*), and the maternally expressed genes *MEG3*, *MEG8*, and anti-sense *RTL1*. *MEG3* and *MEG8* are long intergenic RNAs; *MEG3* has been found dysregulated in several types of tumors, and it is believed to function as a tumor-suppressor gene through interactions with p53 (Zhou et al., 2007). *MEG3* was shown to be downregulated in CML-CP samples, and patients in advanced phase and blast crisis showed further decreased levels of *MEG3* (Zhou et al., 2017). The largest mammalian cluster of microRNAs, together with a cluster of snoRNAs, are included in the maternally expressed strand of this locus. MicroRNAs in this locus were reported as mediators of ground-state pluripotency in mouse embryonic stem cells via inhibition of multi-lineage differentiation and promotion of self-renewal (Moradi et al., 2017), and their expression levels were correlated with pluripotency in mouse induced pluripotent stem cells (Liu et al., 2010). Non-coding RNAs (including microRNAs) from this locus maintained mouse fetal liver and adult long-term repopulating HSCs (LT-HSCs) through the suppression of the PI3K-mTOR pathway, which resulted in inhibition of mitochondrial biogenesis and metabolic activity (Qian et al., 2016). On the other hand, miR-300 belongs to the *DLK1/DIO3* locus and was reported to be highly expressed in quiescent CP and blast crisis CML LSC (CD34^+^CFSE^max^) (Silvestri et al., 2020). We detected a downregulation of microRNAs and snoRNAs from the 14q32 cluster, and the absence of expression of miR-300 in all fractions evaluated by NGS. Both results suggest that the fractions used in our study included mostly non-quiescent CD34^+^CD38^−^ cells with a possible decrease in multipotency. This could also explain the similarity observed between LSC-enriched CD34^+^CD38^−^CD26^+^ and HD-HSC (CD34^+^CD38^−/dim^, expected to include more differentiated cells) fractions (Figures 1A, 2A).

The role of microenvironmental factors in the development of hematological malignancies is an exciting field. In our study, a great number of microRNAs were dysregulated between HSC from CML-CP patients and HSC from HD. This observation could be attributed to cell-autonomous (i.e., genetic or epigenetic alterations) and/or extrinsic factors. Evidence regarding a role of the BM microenvironment in CML include the alteration of the BM niche by LSC by secretion of costimulatory molecules and suppressive cytokines that target metabolic pathways (Zhang et al., 2012; Johnson et al., 2016; Agarwal et al., 2019), the presence of tumor-derived exosomes that can modulate immune responses (Boyiadzis and Whiteside, 2017), and hypoxic conditions that can influence LSC quiescence, differentiation, metabolism and therapy resistance (Ng et al., 2014; Silvestri et al., 2020). It would be of interest to assess whether the miRNome of CML-CP HSC is restored upon TKI treatment.

Bioinformatic tools for microRNA analysis are part of a growing field. Traditional enrichment analysis of pathways based on predicted targets of selected microRNAs resulted in a high rate of false positives. This type of *bias* has been reported as a result of higher redundancy of pathway information at the microRNA level than at gene level (Godard and van Eyll, 2015). Through an overrepresentation analysis that bypasses targets using manually curated lists of microRNA-associations, we detected an enrichment in microRNAs regulated by EGR1 TF, and also in the miR-99b cluster. Interestingly, EGR1 has been reported as regulator of homeostasis of HSC, where it is highly expressed, and downregulated after induction of cell division and migration (Min et al., 2008), whereas miR-99b cluster has been reported as a conserved cluster preferentially expressed in long-term HSC (Guo et al., 2010). By this strategy we also detected an enrichment in lipid metabolism and hematopoiesis (microRNAs upregulated in LSC-enriched vs. CML-CP HSC fractions) and glucose metabolism (microRNAs downregulated in CML-CP HSC vs. HD HSC). In the light of recent descriptions of increased oxidative metabolism in CML LSC-enriched fractions (Kuntz et al., 2017), the results obtained by us serve as a guide for future functional studies that evaluate the role of microRNAs in this process. Kuntz et al. described an increase in fatty acid oxidation and lipolysis, increased glucose oxidation and anaplerosis in stem cell enriched CML fractions, and showed that restriction of mitochondrial functions by treatment with tigecycline had *in vivo* cytotoxic effects on stem/progenitor CML cells, in combination with imatinib (Kuntz et al., 2017). Tigecycline is an FDA-approved antibiotic that inhibits bacterial protein synthesis, but also inhibits the synthesis of mitochondria-encoded proteins. Metabolic vulnerabilities in LSC open the road for new therapeutic strategies, and a thorough understanding of the differential mechanisms involved in CML vs. normal primitive cells is necessary in order to predict possible unfavorable side effects (Vetrie et al., 2020), which have been repeatedly observed in clinical trials that target this population.

## Data Availability Statement

The datasets presented in this study can be found in online repositories. The names of the repository/repositories and accession number(s) can be found below: European Nucleotide Archive, PRJEB41369.

## Ethics Statement

The studies involving human participants were reviewed and approved by Research ethics committee from Instituto Alexander Fleming, Ciudad Autónoma de Buenos Aires, Argentina. The patients/participants provided their written informed consent to participate in this study.

## Author Contributions

MR and MB designed the experiments. MR performed most of the experiments and data analysis. MS contributed to the validation of microRNAs by RT-qPCR and processing of biological samples. SB and CF contributed to small RNA NGS-library construction and sequencing. DK and PY contributed to bioinformatic analysis of NGS-derived data. SC, MC, JF, BM, CP, MP, AV, and VV contributed with patient samples and analysis of clinical data. JS, IZ, IL, and JM participated with helpful discussion and ideas. PV contributed with conceptual input and technical support in CML stem cell experiments, reviewed the data and drafted parts of the manuscript. MR wrote the manuscript. MB supervised the entire work. All authors read and approved the final manuscript.

## Funding

This work was supported by grants from CONICET-FAPERJ (2013), Agencia Nacional de Promoción Científica y Tecnológica of Argentina (PICT-2013 1710), Fundación Mosoteguy, and Fundación SALES. PV and his team are supported by the Austrian Science Fund FWF, grant F4704-B20. MB, IL, PY, and JM are researchers from the Consejo Nacional de Investigaciones Científicas y Tecnológicas of Argentina (CONICET). MR, MS, and DK received CONICET fellowships.

## Conflict of Interest

The authors declare that the research was conducted in the absence of any commercial or financial relationships that could be construed as a potential conflict of interest.

## Abbreviations

BFU-E, burst-forming unit-erythroid; BM, bone marrow; CFU-GEMM, colony-forming unit-granulocyte erythroid macrophage megakaryocyte; CFU-GM, colony-forming unit-granulocyte macrophage; CML, chronic myeloid leukemia; CP, chronic phase; FACS, fluorescent-activated cell sorting; FDA, U.S. Food and Drug Administration; GO, gene ontology; HD, healthy donor; HSC, hematopoietic stem cells; KEGG, kyoto encyclopedia of genes and genomes; LSC, leukemic stem cells; LT-HSC, (murine) long-term repopulating hematopoietic stem cell; MNC, mononuclear cells; NGS, next-generation sequencing; PB, peripheral blood; Ph, Philadelphia chromosome; qPCR, quantitative PCR; RT, reverse transcription; snoRNAs, small nucleolar RNAs; snRNAs, small nuclear RNAs; TKI, tyrosine kinase inhibitors; tRNAs, transfer RNAs.
