# Supplementary material for: miRNome profiling of LSC-enriched CD34+CD38−CD26+ fraction in Ph+ CML-CP samples from Argentinean patients: a potential new pharmacogenomic tool
Source: Front Pharmacol. 2021 Jan 11;11:612573. doi: 10.3389/fphar.2020.612573 (PMC7869017; doi:10.3389/fphar.2020.612573)
Supplement: Supplementary file 4 [file datasheet1.pdf]

Supplementary Material

1 Supplementary Data

1.1 Supplementary results

Assessment of bias in traditional in silico pathway enrichment analysis using lists of small RNAs.

Traditional *in silico* enrichment analysis of both predicted and experimentally validated targets resulted in clusters of functionally related microRNAs that correlated with increased or decreased levels in LSC-enriched $CD34^{+}CD38^{-}CD26^{+}$ vs. putative HSC from CML-CP patients (Sup. Fig. 3). In order to evaluate possible *bias* in the results due to the inclusion of experimentally validated microRNA-mRNA interactions, we performed an identical pathway enrichment analysis using two lists of 16 randomly selected microRNAs, among those that were detectable, but that did not vary (GFOLD=0) among LSC-enriched $CD34^{+}CD38^{-}CD26^{+}$ and CML-CP HSC fractions (see Supplementary Table S3).
Additionally, we performed this analysis using 16 randomly selected microRNAs from each fraction, which resulted in the recurrent inclusion of the majority of KEGG pathways associated to LSC-enriched fractions (Sup. Fig. 4). In order to evaluate whether these recurrent pathways were associated to microRNAs expressed in hematopoietic tissues, we repeated such analysis using four lists of 16 randomly selected microRNAs from a small RNA-seq experiment performed with neuroblastoma cells (Array Express E-GEOD-72721)(Hsu et al., 2016). Surprisingly, we observed a high overlap of enriched pathways with those detected in hematopoietic fractions (>45%, Sup. Fig. 4).

1.2 Supplementary methods

Red cell lysis of mononuclear cells
Cells were resuspended in 1mL of pre-warmed (37°C) red cell lysis solution: ethylenediaminetetraacetic acid (EDTA) 0.13mM, potassium bicarbonate ($KHCO_3$) 1mM, ammonium chloride (NH_4Cl) 170mM, pH 7.3, and vigorously mixed for 1 minute. After addition of 9mL of red cell lysis solution, cells were incubated 3 minutes at 37°C. Cells were washed by addition of 30mL of phosphate buffered saline (PBS) and centrifuged.

Isolation of $CD34^{+}$ cells
Cells were incubated with 100 µL of beads (CD34 MicroBeads, Miltenyi Biotech), and 100 µL of FcR-block for 30 minutes, for every $1x10^8$ MNC (in ~300 µL of ice-cold MACS buffer), in a water bath at 2-8°C. All subsequent steps were performed with cold MACS buffer (Bovine Serum Albumin 0.5%, EDTA 2mM, PBS, pH 7.2): cells were washed twice (4 mL), then seeded in a MS column (Miltenyi Biotech) in a volume of 500 µL, washed four times (1x 1 mL, 3 x 500 µL), and eluted in a 2 mL volume. Cells were immediately incubated with antibodies for FACS sorting, or freezed in 1mL of cold freezing medium: Dulbecco's Modified Eagle Medium (DMEM, GIBCO) 50%/ Human Serum Albumin 40%/ Dimethyl sulfoxide 10%. During thawing of $CD34^{+}$ cells, cells were quickly thawed in a water bath at 37°C, diluted in 30mL of Roswell Park Memorial Institute 1640 medium (RPMI 1640, GIBCO), centrifuged, resuspended in 50-100 µL of MACS buffer, counted and immediately incubated with antibodies for FACS sorting.

Assessment of purity in sorted fractions.

Total RNA was extracted from CFU assay-derived colonies following manufacturer's instructions (RNAqueous-Micro Kit, Ambion). RNA was eluted in 18 µL of pre-warmed (75°C) elution solution, and reverse transcribed using Superscript II enzyme (Invitrogen) and random primers (final concentration: 20 ng/µL, Invitrogen), in a reaction volume of 25 µL, following manufacturer's instructions. cDNA was diluted (1/2) with distilled water, and kept at -20°C. Detection of *BCR-ABL1* and *ABL1* was performed by qPCR using primers (final concentration: 300 nM each, Invitrogen) and probes (final concentration: 200nM, SIGMA-Aldrich) recommended by the "Europe against cancer" program, Taqman Universal MasterMix II, no UNG (Applied Biosystems), by means of an Applied Biosystems 7500 Real-time PCR machine. Absolute copy numbers and amplification efficiency were estimated by the inclusion of calibration curves built with standardized plasmids (BCR-ABL pDNA calibrant; ERM certified reference material ERMAD623, SIGMA).

Primers used for quantification of microRNAs by RT-qPCR

Stem-loop primers for reverse transcription of microRNAs	
hsa-miR-92b-3p RT	GTCGTATCCAGTGCAGGGTCCGAGGTATTCGCACTGGATACGACGGAGGC
hsa-miR-125a-5p RT	GTCGTATCCAGTGCAGGGTCCGAGGTATTCGCACTGGATACGACTCACAG
hsa-miR-182-5p RT	GTCGTATCCAGTGCAGGGTCCGAGGTATTCGCACTGGATACGACAGTGTG
novel-3 RT	GTCGTATCCAGTGCAGGGTCCGAGGTATTCGCACTGGATACGACCAAGAC
hsa-miR-2355-5p RT	GTCGTATCCAGTGCAGGGTCCGAGGTATTCGCACTGGATACGACTTGTCC
hsa-miR-126-5p RT	GTCGTATCCAGTGCAGGGTCCGAGGTATTCGCACTGGATACGACCGCGTA
hsa-miR-132-3p RT	GTCGTATCCAGTGCAGGGTCCGAGGTATTCGCACTGGATACGACCGACCA
hsa-let-7a-5p RT	GTCGTATCCAGTGCAGGGTCCGAGGTATTCGCACTGGATACGACTTCTAT
hsa-miR-10a-5p RT	GTCGTATCCAGTGCAGGGTCCGAGGTATTCGCACTGGATACGACCACAAA
hsa-miR-196a-5p RT	GTCGTATCCAGTGCAGGGTCCGAGGTATTCGCACTGGATACGACCCCAAC

Forward primers for qPCR	
hsa-miR-92b-3p Fw	TGCATTTCTATTGCACTCGTC
hsa-miR-125a-5p Fw	TGATCCCTGAGACCCTTTAAC
hsa-miR-182-5p Fw	GGTTTGGCAATGGTAGAACT
novel-3 Fw	CGGACTGAAGGTAGATAGAACAG
hsa-miR-2355-5p Fw	TCGAAGATCCCCAGATACAAT
hsa-miR-126-5p Fw	CAGCGGCATTATTACTTTTGG
hsa-miR-132-3p Fw	CTCGGTAACAGTCTACAGCCA
hsa-let-7a-5p Fw	GCGGTGAGGTAGTAGGTTGT
hsa-miR-10a-5p Fw	GGCTACCCTGTAGATCCGAA
hsa-miR-196a-5p Fw	GCGTCGTAGGTAGTTTCATGTT
snRNA U6 Fw	GCTTCGGCAGCACATATACTAAAAT
Reverse primers for qPCR	
Universal Rv (microRNAs)	GTGCAGGGTCCGAGGT
snRNA U6 Rv	CGCTTCACGAATTTGCGTGTCAT

2 Supplementary Figures and Tables

2.1 Supplementary figures.

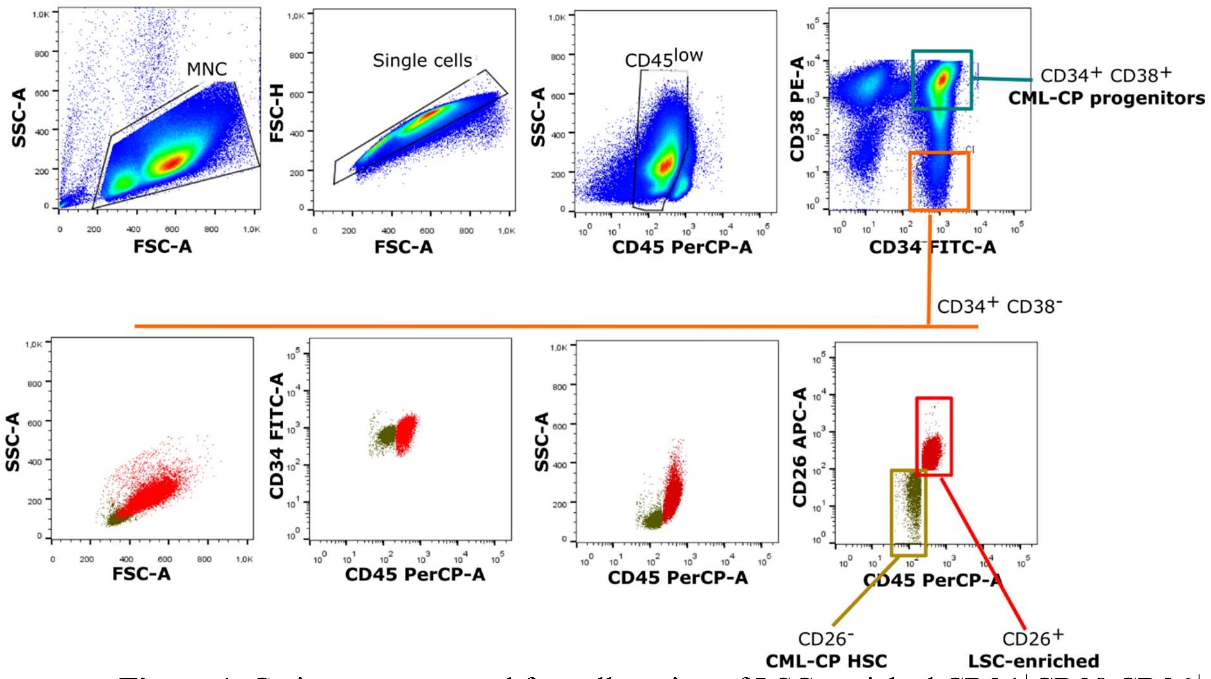

Supplementary Figure 1. Gating strategy used for cell sorting of LSC-enriched $CD34^{+}CD38^{-}CD26^{+}$ and HSC fractions from BM or PB CML-CP patients at diagnosis.

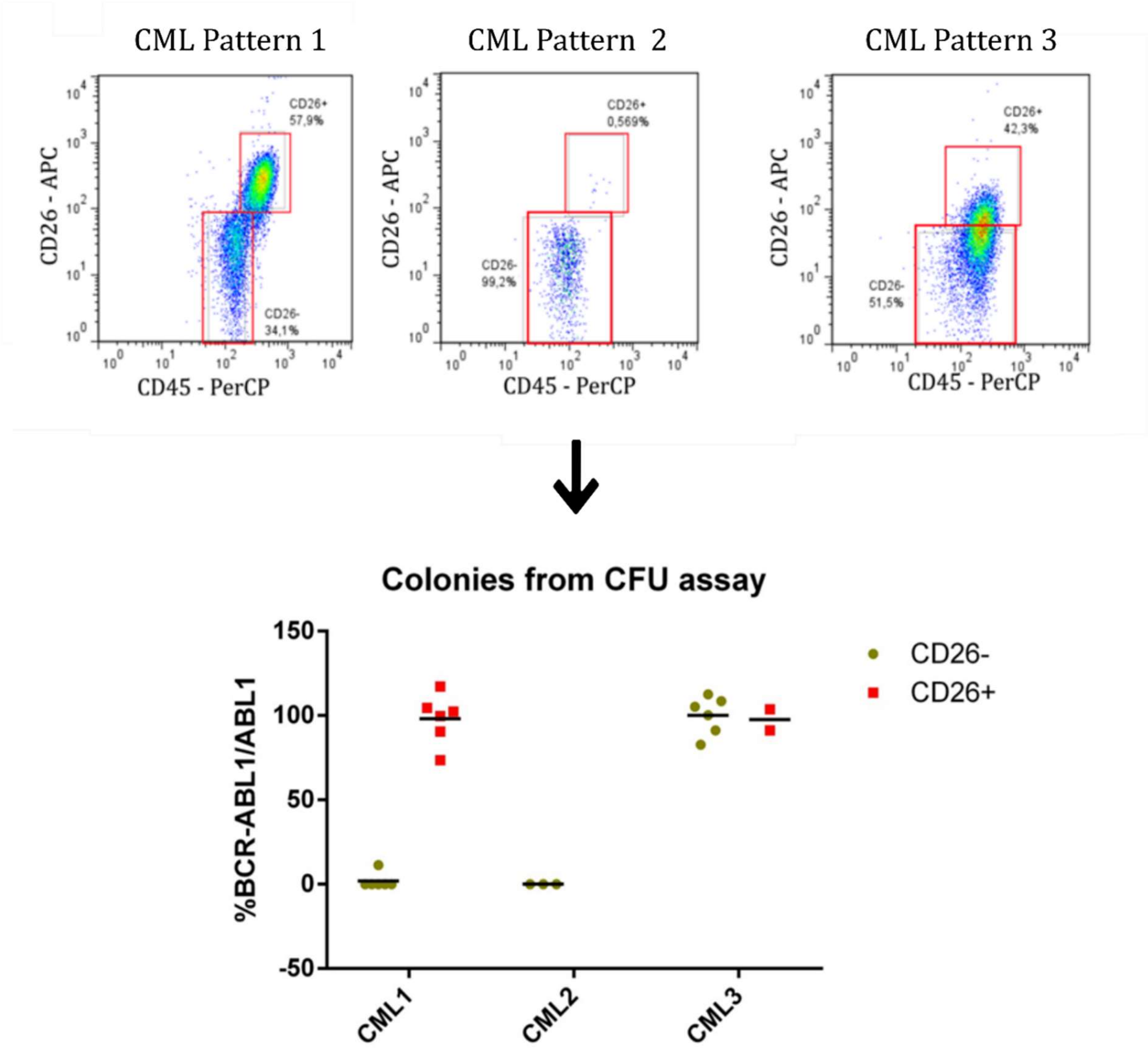

Supplementary Figure 2. Gating Representative flow cytometry patterns observed in $CD34^{+}CD38^{-}$ fractions from CML-CP patients at diagnosis. Pattern 1 showed coexistence of both LSC-enriched $CD34^{+}CD38^{-}CD26^{+}$ and HSC populations; pattern 2 showed mostly HSC; pattern 3 showed a single population with varying levels of CD26. Assessment of purity of each fraction revealed that $CD26^{-}$ cells in pattern 3 expressed *BCR-ABL1* mRNA and were not further used for microRNA isolation. Bottom: Each dot represents a pool of 4-6 colonies. CML1, CML2 and CML3 correspond to three single patients with flow cytometry patterns 1, 2 or 3, respectively.

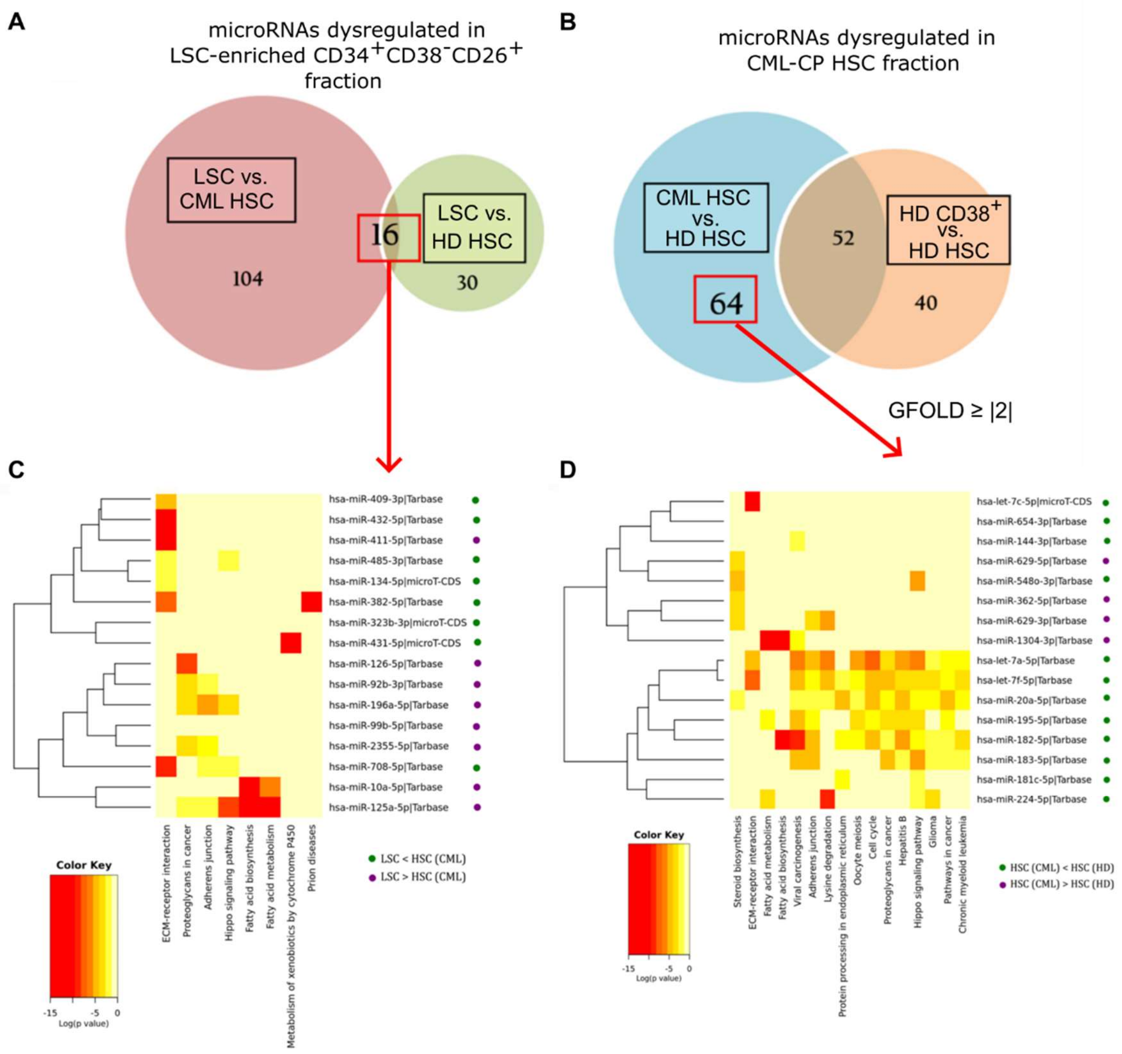

Supplementary Figure 3. Differential expression and pathway analysis of small-RNA NGS data by miRPath (*DIANA tools*). A, B: Number of microRNAs with GFOLD≥|1| in each comparison. Selected microRNAs are indicated in red. C, D: Heatmaps of KEGG molecular pathways associated with predicted and experimentally validated targets of microRNAs dysregulated in LSC-enriched $CD34^{+}CD38^{-}CD26^{+}$ (C: pathway union) or HSC fractions from CML-CP patients (D:pathway union). Green and purple dots refer to the relative abundance of each microRNA in each population.

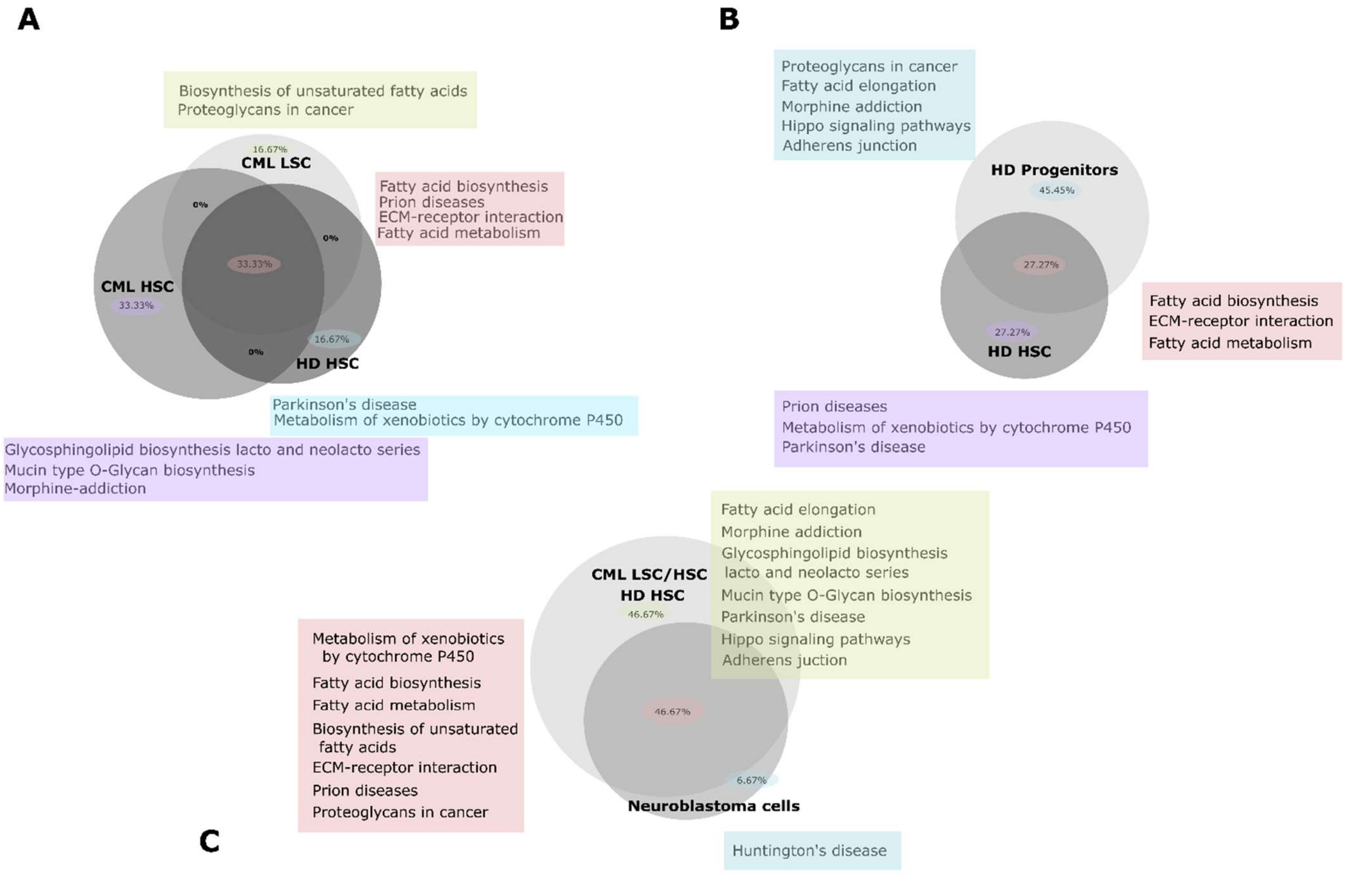

Supplementary Figure 4. Venn diagrams of KEGG molecular pathways (miRPath, pathway union) associated with predicted and experimentally validated targets of microRNAs randomly selected in LSC-enriched $CD34^{+}CD38^{-}CD26^{+}$, CML-CP HSC and HD HSC fractions (A), or HSC and progenitor fractions from HD (B). All pathways shown in A and B were compared with KEGG pathways associated with predicted and experimentally validated targets of microRNAs randomly selected from neuroblastoma cells (Array Express E-GEOD-72721) (C).

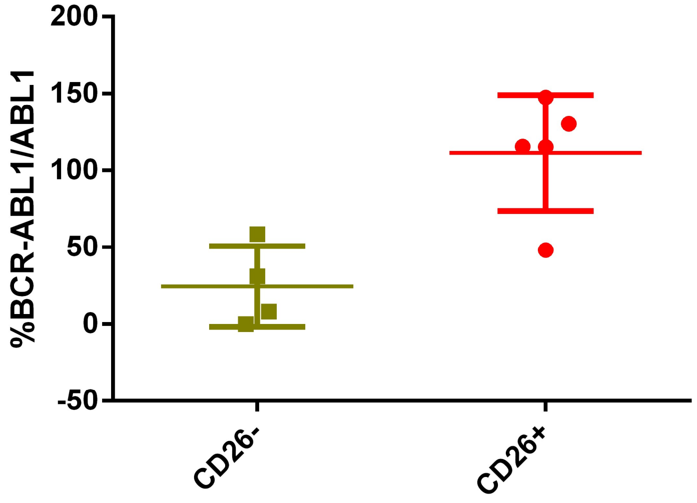

Supplementary Figure 5. Assessment of purity by detection of *BCR-ABL1* mRNA in sorted fractions used for validation (RT-qPCR in a new cohort of samples). Each dot represents a different fraction from all patients evaluated.

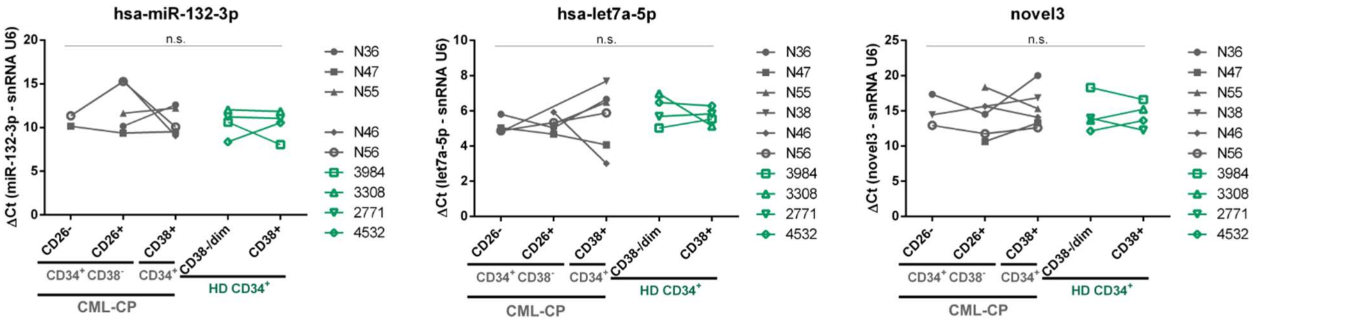

Supplementary Figure 6. Validation of microRNAs by RT-qPCR in a new cohort of CML-CP and HD samples: results for miR-132-3p, let7a-5p, and "novel-3". Results are expressed as ΔCt = Ct (microRNA) - Ct (snRNA U6). Each dot is the mean of technical duplicates from each patient or HD. CML-CP samples are represented in grey symbols, and HD samples in green symbols. Lines connect different fractions from the same patient or HD. n.s.= not statistically significant differences (linear mixed-effects model, global $\alpha = 0.05$)

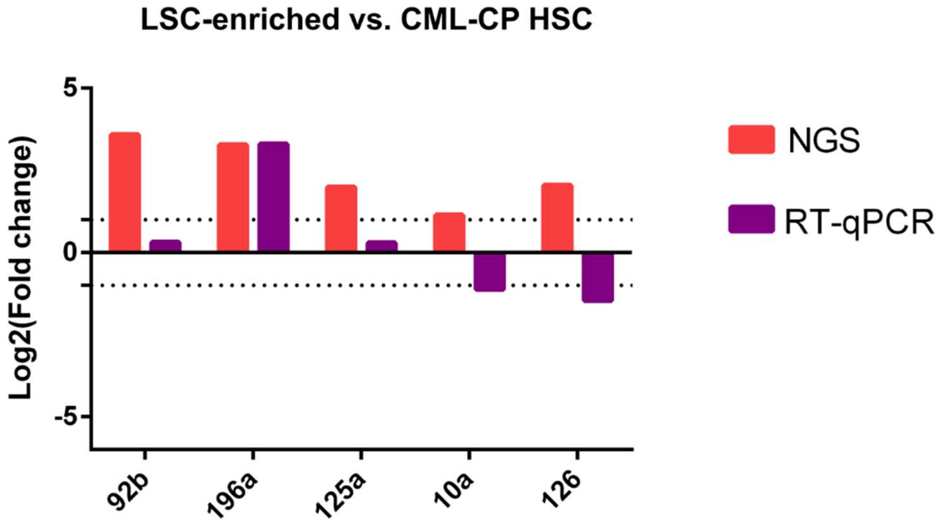

Supplementary Figure 7. Comparison of NGS and RT-qPCR results. Mean Log_2(Fold change) was calculated between LSC-enriched $CD34^+CD38^-CD26^+$ and CML-CP HSC fractions. Dotted line indicates Log_2(Fold change) =1 or -1.

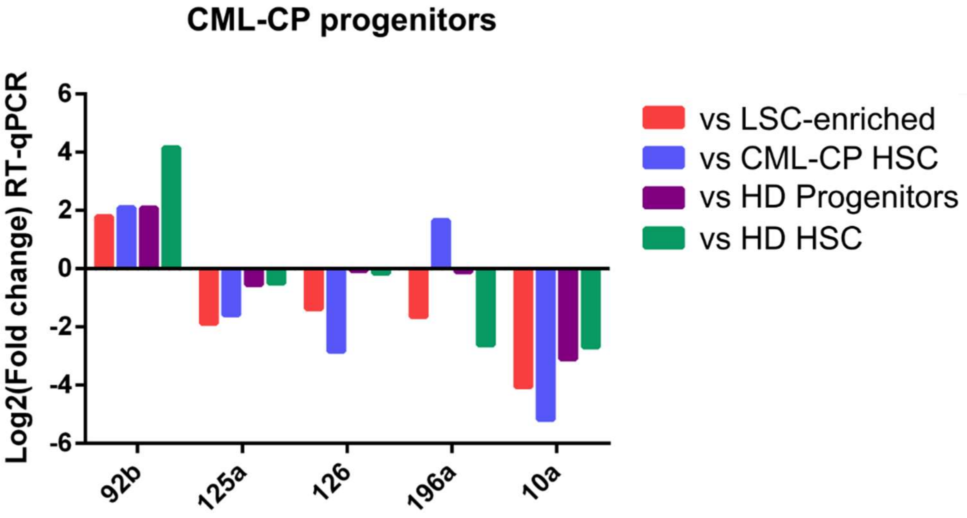

Supplementary Figure 8. Mean Log_2(fold change) values for all samples evaluated by RT-qPCR between CML-CP progenitors ($CD34^+CD38^+$) and LSC-enriched $CD34^+CD38^-CD26^+$ fraction (red), CML-CP HSC fraction (blue), HD progenitors (purple) and HD HSC (green), for each microRNA.

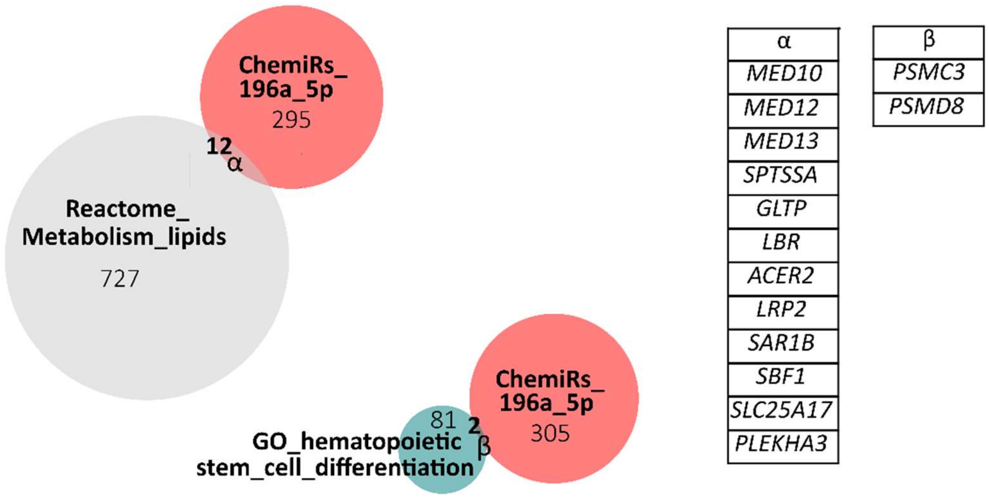

Supplementary Figure 9. Search for possible targets of miR-196a-5p as the intersection of experimentally validated targets reported in ChemiRs, genes included under the category of "Metabolism of lipids" in Reactome, or the category "Hematopoietic stem cell differentiation" in Gene Ontology. Genes included in the intersections are detailed.

2.2 Supplementary tables

Supplementary table S1. Experimental design of multiplex RT of microRNAs performed during validation by RT-qPCR.

Multiplex 1 (M1)	Multiplex 2 (M2)
miR-92b-3p<br>miR-125a-5p<br>miR-182-5p<br>novel-3<br>miR-2355-5p<br>miR-126-5p	miR-132-3p<br>let-7a-5p<br>miR-10a-5p<br>miR-196a-5p
Positive control: KU812	Positive control: TF1a

RT of microRNAs was performed on two multiplex reactions (M1 and M2), each including different combinations of stem-loop primers. KU812 and TF1a cell lines were used as positive controls.

Supplementary table S2. MicroRNAs dysregulated in LSC-enriched $CD34^{+}CD38^{-}CD26^{+}$ fraction by small RNA-NGS.

	GFOLD value		DESeq normalised counts		
	**CML-CP HSC vs LSC-enriched fractions**	**HD HSC vs LSC-enriched fractions**	**LSC-enriched**	**HSC (CML-CP)**	**HSC (HD)**
hsa-mir-92b-3p	-3.6	1.6	2062	207	5951
hsa-mir-196a-5p	-3.6	-2.7	135	6	13
hsa-mir-126-5p	-2.0	1.1	18667	5343	44262
hsa-mir-125a-5p	-1.9	-1.0	6225	2114	3045
hsa-mir-2355-5p	-1.5	-1.0	928	385	444
hsa-mir-99b-5p	-1.2	-1.7	3907	2214	1207
hsa-mir-411-5p	-1.2	3.6	36	9	749
hsa-mir-10a-5p	-1.1	1.1	28770	19727	68583
hsa-mir-708-5p	1.6	2.5	4	46	42
hsa-mir-431-5p	1.6	1.9	8	112	89
hsa-mir-134-5p	1.9	2.5	1	64	62
hsa-mir-485-3p	2.2	2.7	1	53	46
hsa-mir-409-3p	2.6	3.8	46	595	902
hsa-mir-323b-3p	2.8	2.2	3	140	60
hsa-mir-432-5p	2.9	2.4	0	105	65
hsa-mir-382-5p	3.7	2.9	3	130	52

GFOLD can be considered as a robust Log_2(fold change) value. GFOLD<0: increased in LSC-enriched $CD34^{+}CD38^{-}CD26^{+}$ fraction; GFOLD>0: decreased in LSC-enriched $CD34^{+}CD38^{-}CD26^{+}$ fraction.

Supplementary table S3. Molecular pathways associated to targets of selected microRNAs with detectable counts and GFOLD=0 between LSC-enriched $CD34^{+}CD38^{-}CD26^{+}$ and CML-CP HSC fractions.

List 1: hsa-mir-3136, hsa-mir-543, hsa-mir-3202, hsa-mir-1256, hsa-mir-1976, hsa-mir-1185-1-3p, hsa-mir-639, hsa-mir-550a-3p, hsa-mir-548b-5p, hsa-mir-3136-5p, hsa-mir-939-5p, hsa-mir-3134 hsa-mir-375, hsa-mir-1908-5p, hsa-mir-202-5p, hsa-mir-885-5p			
KEGG pathway	p-value	#genes	#microRNAs
Fatty acid biosynthesis	7.77E-16	1	1
List 2: hsa-mir-612, hsa-mir-650, hsa-mir-632, hsa-mir-564, hsa-mir-1225-5p, hsa-mir-135a-5p, hsa-mir-3140-3p, hsa-mir-3200-3p. hsa-mir-1909-5p, hsa-mir-760, hsa-mir-3144-3p, hsa-mir-3125, hsa-mir-3161, hsa-mir-3121-3p, hsa-mir-611, hsa-mir-1252-5p			
KEGG pathway	p-value	#genes	#microRNAs
Extracellular matrix-receptor interaction	0	22	3
Hippo signaling pathway	2.21E-01	7	2

.

Supplementary table S4. snoRNAs dysregulated in LSC-enriched $CD34^{+}CD38^{-}CD26^{+}$ fraction by small RNA-NGS.

	GFOLD value	
	**HD HSC vs LSC-enriched fraction**	**HD HSC vs. CML-CP HSC fraction**
14q(II-24)	0.6	1.1
14q(II-21)	1.0	0
14q(II-15)	1.1	1
14q(II-22)	3.4	2.5
14q(II-9)	3.5	2.2

GFOLD can be considered as a robust Log_2(fold change) value. HD HSC vs LSC-enriched $CD34^{+}CD38^{-}CD26^{+}$ fraction: GFOLD<0: increased in LSC-enriched, GFOLD>0: decreased in LSC-enriched fraction. HD HSC vs CML-CP HSC: GFOLD<0: increased in CML-CP HSC, GFOLD>0: decreased in CML-CP HSC fraction.

Supplementary table S5. MicroRNAs dysregulated in CML-CP HSC by small RNA-NGS.

	GFOLD	DESeq normalised counts	
	**HD HSC vs CML-CP HSC**	**HSC (CML-CP)**	**HSC (HD)**
hsa-mir-1304-3p	-3.6	1179	765
hsa-mir-629-5p	-2.3	2841	421
hsa-mir-629-3p	-2.3	1431	167
hsa-mir-362-5p	-2.0	7186	1204
hsa-mir-20a-5p	2.0	4054	16670
hsa-mir-195-5p	2.1	16	104
hsa-mir-654-3p	2.4	67	516
hsa-mir-224-5p	2.5	2	36
hsa-mir-144-3p	2.6	415	3244
hsa-mir-181c-5p	2.7	2315	12831
hsa-let-7c-5p	2.7	236	896
hsa-let-7f-5p	2.9	23477	139037
hsa-mir-548o-3p	3.0	11	157
hsa-let-7a-5p	3.0	17636	120313
hsa-mir-182-5p	3.5	4567	41803
hsa-mir-183-5p	3.8	328	4196

GFOLD can be considered as a robust Log_2(fold change) value. GFOLD<0: increased in CML-CP HSC; GFOLD>0: decreased in CML-CP HSC.
